# Use of acute anti-migraine medication and risk of development of chronic headache: a prospective population based study

**DOI:** 10.1186/1129-2377-14-S1-P164

**Published:** 2013-02-21

**Authors:** S Schramm, MS Yoon, A Katsarova, M Obermann, G Fritsche, S Moebus, Z Katsarava

**Affiliations:** 1 Institute of Medical Informatics, Biometry and Epidemiology, University Duisburg-Essen, Germany; 2 Department of Neurology, Ruhr-University Bochum, Germany; 3 Department of Neurology and Headache Center, University Duisburg-Essen, Germany; 4 Department of Neurology, Evangelisches Krankenhaus Unna, Germany

## Introduction

Overuse of acute pain medication is a risk factor for developing chronic headache.

## Objectives

To investigate the association between the use of acute anti-migraine medication as single analgesics (SA), combination analgesics (CA) and triptanes (T) and the risk for chronic headache in patients with episodic migraine.

## Methods

We used data of the German Headache Consortium (GHC) Study which is a population-based sample of 18,000 participants aged 18 to 65 years. Information about headache features, frequency, use of medication and years of education were collected at baseline (2003-2005) and follow up one (t1) and two (t2) years after baseline using mailed questionnaires. Participants with prophylactic headache or other prophylactic pain medication were excluded (n=209). Primary outcome was defined as incidence of chronic headache (any headache on ≥15 days/month) at t1 or t2 in participants with episodic migraine (≤14 days of migraine/month) at baseline. We estimated odds ratios (OR) and 95%-confidence intervals (95%-CI), adjusting for headache days at baseline (interval scaled), education, age (interval scaled), gender and BMI classes (normal, overweight, obese).

## Results

Of 18,000 people 9,944 (55.2%) responded at baseline, of those 6,688 (67.3%) resp. 6,975 (70.1%) responded at t1 resp. t2. At baseline 1,601 participants had episodic migraine. The incidence of chronicity was 6.2%. Use of anti-migraine medication had a protective effect compared to no intake (SA: OR=0.39, 95%-CI=0.19-0.78; CA: 0.60, 0.22-1.61; T: 0.34, 0.10-1.15). This effect was stronger for SA than for CA (OR=0.65, 95%-CI=0.28-1.50). Adjusting for age, gender and BMI classes did not notably change these results.

## Conclusion

Our data indicate that use of acute anti-migraine medication irrespective of the type (SA, CA, T) reduces the risk for developing chronic headache.

## Conflict of interest

Z. Katsarava received speaker honoraria and/or travel reimbursement from Allergan, Merck Serono, Bayer Shering, Biogen, St Jude Medical and served as consultant to Allergan.
